# Accuracy of tympanic temperature measurement using an infrared tympanic membrane thermometer

**DOI:** 10.1186/1756-0500-6-194

**Published:** 2013-05-10

**Authors:** Gasim I Gasim, Imad R Musa, Mohamed T Abdien, Ishag Adam

**Affiliations:** 1 College of Medicine, Qassim University, Qassim, Saudi Arabia; 2 Buraidah Central Hospital, Buraidah, Saudi Arabia; 3 Faculty of Medicine, University of Khartoum, P.O. Box 102, Khartoum, Sudan

**Keywords:** Tympanic membrane, Axillary temperature, Sudan, Infrared, Thermometry

## Abstract

**Background:**

During investigation and diagnosis of patients, accurate temperature measurement is of great importance. The advantages of tympanic membrane thermometry are speed (temperature reading available within seconds), safety, and ease of use. The aim of this study was to compare the accuracy of infrared tympanic thermometers in comparison to mercury thermometers in measurement of body temperature.

**Methods:**

Axillary and tympanic temperature was measured simultaneously in consecutive patients using mercury glass and infrared tympanic thermometers at Omdurman Hospital, Sudan during October 2012.

**Results:**

In total, temperature was measured in 174 patients, 95 of whom (54.6%) were male. The mean (SD) patient age and weight was 33.18 (25.07) years and 52.13 (69.85) kg. There was no significant difference in mean (SD) temperature measurement between mercury and infrared tympanic membrane thermometers, 37.29°C (0.91) versus 37.38°C (0.95), *P* = 0.373, respectively. There was a significant positive correlation between axillary and tympanic body temperature measurements (*r* = 0.697, *P* < 0.001). The mean difference between the two readings (with limits of agreements) was - 0.093 (−0.20; 0.02) °C.

**Conclusion:**

In this study, tympanic membrane thermometry is as reliable and accurate as axillary mercury glass thermometry. Thus, tympanic thermometry can be used in clinical practice, especially in the emergency setting, where ease of use and speed of obtaining the temperature reading are important.

## Background

Measurement of body temperature is one of the oldest known diagnostic methods and still remains an important indicator of health and disease, both in everyday life and in medical care [1]. Sudan has endemic levels of many communicable diseases, such as malaria [2,3], which necessitate speedy, safe and accurate temperature measurement for screening for fever.

Body temperature depends on the type of thermometer and the area of the body used for taking the measurement [4]. Human body temperature varies depending on the site from which the reading was taken - these differences are actually no more than an approximation of the true value that is being estimated [4,5]. Integration of thermal inputs occurs at numerous levels within the central nervous system, involving the spinal cord, neurons in the midbrain, reticular formation, and vagus, finally arriving at the hypothalamus which is the master thermoregulatory controller in mammals. Autonomic thermoregulatory control has five main contributors, the skin surface, deep abdominal and thoracic tissues, the spinal cord, the hypothalamus and other portions of the brain [6]. Although the temperature measured by indwelling pulmonary artery catheters represents core body temperature [7], this method is invasive and unsuitable for most patients. Axillary thermometry is a non-invasive technique which reflects the body temperature and correlates with the rectal temperature [8]. The use of mercury thermometers, especially glass mercury thermometers is not without hazard [9].

Infrared tympanic membrane thermometers are considered ideal because the tympanic membrane and the hypothalamus share an arterial blood supply originating from the carotid artery; therefore, the tympanic membrane is considered to directly reflect core temperature [10]. An infrared tympanic membrane thermometer is easy to use and is favored over a conventional mercury thermometer provided its accuracy is guaranteed [11,12]. Few published data are available on the accuracy of tympanic membrane thermometers. Thus, the aim of the current study was to compare the temperature obtained by tympanic membrane thermometers with that obtained with mercury glass thermometers before recommending tympanic thermometers for use in general practice in Sudan.

## Methods

### Patient selection

This study was conducted at Omdurman Teaching Hospital, Sudan during October 2012. Patients including both adults and children above one year of age, presenting with or without fever to the emergency room during this period were evaluated for inclusion in the study. Patients were recruited by convenience sampling (based on a 2-sided hypothesis tests using Epiinfo with 80% power and confidence interval of 95%) until 174 sets of simultaneous axilla and tympanic membrane temperature measurements were obtained. Those with otitis externa/media, soft tissue infection, severe illness, trauma patients, those who had had a cold or hot drink or who had smoked in the 20 minutes prior to examination, those wearing a hearing aid and those who were unwilling to be enrolled in the study were excluded.

The study was approved by the ethical committee at Omdurman Hospital, and written consent was obtained from all patients or from a guardian for children.

### Instruments

A Braun ThermoScan (IRT 4520, Braun GmbH, Kronberg, Germany), a device validated in a previous study [13], was used for the infrared measurement of tympanic membrane temperature. A non-self-adjusted mercury bulb thermometer was used to measure axillary temperature. All the mercury bulb thermometers were calibrated in a single water bath set at 38°C. Only those thermometers with a deviation of less than 0.1°C were used for the study. All patients were examined otoscopically to exclude ear infection and occluding ear wax was cleared. The same procedure using the right ear right was used to measure the tympanic membrane temperature for each patient.

### Procedure

A medical officer and three nurses received training on the proper use of all temperature measuring devices. Their visual acuity in both eyes tested 6/6. Patients who fulfilled the study criteria had their axilla and tympanic membrane temperatures simultaneously measured at 08.00 h. The probe of the infrared thermometer was inserted into the external auditory meatus by pulling the pinna backward, and directing the probe towards the eye. The probe was held in the same position until the beep was heard. The mercury bulb thermometer was shaken before each recording to decrease its temperature reading to below 35°C and then placed, for a minimum of 5 minutes, in the patient’s axilla. The same healthcare worker would read and document the digital reading from the tympanic membrane thermometer and the mercury bulb thermometer. Immediately, another member of the team would then read and document the mercury thermometer, blinded from the results of the original healthcare worker.

### Statistical analyses

The results were analyzed using SPSS, version 20.0 for Windows (SPSS Inc, Chicago, IL, USA). Linear correlations were made between tympanic and axillary temperatures. Differences between sets of data were plotted as described by Bland-Altman [14]. Based on previously pre-defined clinically acceptable limits, agreement between tympanic and axillary measurement methods was accepted when the mean ± 2 standard deviations was within ± 0.2°C [15].

## Result

### General characteristics

Temperature was measured for 174 patients (67; 38.5% were children < 18 years), all of whom were medical cases. There were 95 (54.60%) males, their mean age (SD) was 30.1 (24.1) years (range 2–80 years) and mean weight (SD) was 54.4 (69.8) kg. likewise for the females, the mean age (SD) was 32.4 years (range 2–80) and the mean weight (SD) was 56.1(62.7) kg. Out of these 174 patients, 61 (35.1%) patients were febrile (temperature >37.5°C) according to both axillary and tympanic readings.

### Axillary body temperature versus tympanic body temperature

The mean (SD) body temperature measurements were 37.29 (0.91; axillary) and 37.38 (0.95; tympanic), *P* = 0.373°C (Table 1).

**Table 1:** Comparison of axillary and tympanic body temperature measurement (°C)

**Method of measurement**	**Mean ± SD**	**Median**	**(min; max)**	**P**
**Axillary temperature**	37.29 ± 0.91	37.20	(35.50; 40.70)	0.373
**Tympanic temperature**	37.38 ± 0.95	37.25	(35.20; 40.40)	

**Figure 1:**
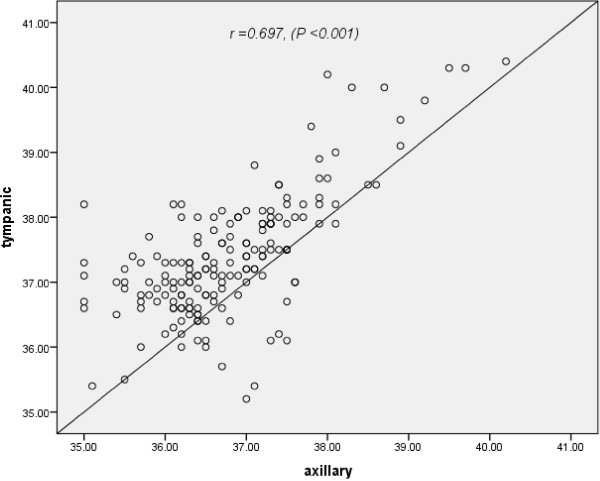
Scatter plot of correlation between body temperature measurements by infrared tympanic and mercury glass thermometers.

**Figure 2:**
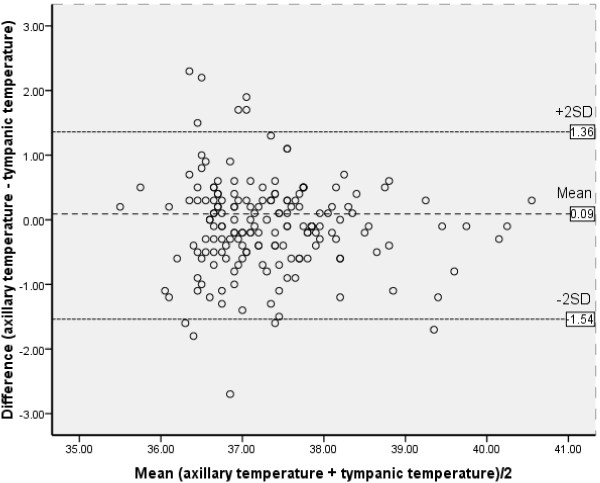
Bland-Altman plot of the differences between infrared tympanic thermometry and mercury glass temperature measurements.

There was a positive correlation between body temperature using axillary and tympanic methods (*r* = 0.697, *P* < 0.001; Figure 1). The mean difference, with limits of agreements, between the two readings was −0.093 (−0.20; 0.02) °C (Table 2 and Figure 2).

**Table 2:** Correlation, bias, and limits of agreement between axillary and tympanic measurements of body temperature

**Axillary vs. tympanic temperature**	**Correlation Coefficient**	**Bias ± SD**	**95% CI**	**Limits of agreement**
	0.697	−0.093 ± 0.72	−0.20; 0.02	−1.54 to 1.36

## Discussion

The main finding of the current study was a positive correlation between axillary and tympanic methods of measuring body temperature. This study positively answered the question ‘How well does tympanic membrane temperature measurement agree with standard axillary measuring technique?’, or can the new device substitute for the old? Infrared tympanic membrane thermometers take seconds to measure the natural emission of infrared thermal radiation from the tympanic membrane. However, doubts have been expressed about the accuracy of tympanic membrane thermometry [16,17] and differences have been observed when measurements are made in both ears. Ear infections e.g. otitis media can influence the true temperature of the tympanum [18]. A further study comparing rectal temperature measurement with infrared tympanic thermometer measurement did not find excellent agreement of results [19]. Similar results to the present study were obtained by Chue *et al.,* 2012 who compared tympanic and oral mercury thermometers in 201 patients on the Thai-Myanmar border, and found the mean difference in the two devices for all observers/devices to be 0.09°C (95% CI: 0.07–0.12) [10]. More than one reading was used by Chue *et al.*, and only one reading was used in the current study. Likewise, Rabbani *et al.,* 2010 reached the same conclusion, especially in young patients, where both tympanic membrane and oral cavity temperatures were recorded, as well as oral temperature as standard [20]. However, Edelu *et al.,* 2011 compared infrared tympanic thermometer in oral mode with mercury glass thermometer readings for measuring the temperature in febrile and afebrile children less than 5 years old, and found a mean difference of 0.41 ± 0.37°C (*P* < 0.001) in the febrile group and 0.47 ± 0.39°C (*P* < 0.001) in the afebrile group. Although tympanic membrane thermometers have a fairly good sensitivity and specificity, the study concluded that they may not be reliable in estimating ‘core’ body temperature in children [21].

Findings from the present study support the use of infrared tympanic membrane thermometers, because of their safety, ease of use and the fast speed for obtaining temperature readings. Moreover, the hazards of mercury toxicity makes infrared thermometers preferable to the old mercury glass thermometers, although some might prefer the latter based on their low cost compared with tympanic membrane thermometers.

One of the limitations of the present study was the use of axillary temperature as a measurement of core temperature rather than the rectal one. Axillary temperature is easy to use, commonly used in our setting and might be acceptable by the traditions and customs in this setting. Pulmonary artery temperature correlates best with core temperature, but to measure this requires an invasive procedure which is unsuitable for use in routine emergency care practice. Furthermore, this study did not include patients with hypothermia, and so its findings cannot be extrapolated to newborns or to patients with hypothermia. Further studies including these groups should be carried out to support the wider use of tympanic membrane thermometers.

## Conclusion

In this study, tympanic membrane thermometry was as reliable and as accurate as axillary mercury glass thermometry. Thus, tympanic membrane thermometry can be used in the clinical practice, because it is easy to use and the speed of obtaining the temperature reading.

## Competing interests

The authors declare that they have no competing interests.

## Authors’ contributions

GIG and IA designed the study. ERM and MTA supervised the temperature measurements and completed the statistical analyses. All the authors helped draft and have approved the final version of the paper.
